# Cell entry mechanisms of SARS-CoV-2

**DOI:** 10.1073/pnas.2003138117

**Published:** 2020-05-06

**Authors:** Jian Shang, Yushun Wan, Chuming Luo, Gang Ye, Qibin Geng, Ashley Auerbach, Fang Li

**Affiliations:** ^a^Department of Veterinary and Biomedical Sciences, College of Veterinary Medicine, University of Minnesota, Saint Paul, MN 55108

**Keywords:** COVID-19, SARS-CoV-2, SARS-CoV, ACE2 receptor, proprotein convertase furin

## Abstract

A novel severe acute respiratory syndrome (SARS)-like coronavirus (SARS-CoV-2) is causing the global coronavirus disease 2019 (COVID-19) pandemic. Understanding how SARS-CoV-2 enters human cells is a high priority for deciphering its mystery and curbing its spread. A virus surface spike protein mediates SARS-CoV-2 entry into cells. To fulfill its function, SARS-CoV-2 spike binds to its receptor human ACE2 (hACE2) through its receptor-binding domain (RBD) and is proteolytically activated by human proteases. Here we investigated receptor binding and protease activation of SARS-CoV-2 spike using biochemical and pseudovirus entry assays. Our findings have identified key cell entry mechanisms of SARS-CoV-2. First, SARS-CoV-2 RBD has higher hACE2 binding affinity than SARS-CoV RBD, supporting efficient cell entry. Second, paradoxically, the hACE2 binding affinity of the entire SARS-CoV-2 spike is comparable to or lower than that of SARS-CoV spike, suggesting that SARS-CoV-2 RBD, albeit more potent, is less exposed than SARS-CoV RBD. Third, unlike SARS-CoV, cell entry of SARS-CoV-2 is preactivated by proprotein convertase furin, reducing its dependence on target cell proteases for entry. The high hACE2 binding affinity of the RBD, furin preactivation of the spike, and hidden RBD in the spike potentially allow SARS-CoV-2 to maintain efficient cell entry while evading immune surveillance. These features may contribute to the wide spread of the virus. Successful intervention strategies must target both the potency of SARS-CoV-2 and its evasiveness.

## Significance

A key to curbing SARS-CoV-2 is to understand how it enters cells. SARS-CoV-2 and SARS-CoV both use human ACE2 as entry receptor and human proteases as entry activators. Using biochemical and pseudovirus entry assays and SARS-CoV as a comparison, we have identified key cell entry mechanisms of SARS-CoV-2 that potentially contribute to the immune evasion, cell infectivity, and wide spread of the virus. This study also clarifies conflicting reports from recent studies on cell entry of SARS-CoV-2. Finally, by highlighting the potency and the evasiveness of SARS-CoV-2, the study provides insight into intervention strategies that target its cell entry mechanisms.

The emergence and rapid spread of a novel severe acute respiratory syndrome (SARS)-like coronavirus SARS-CoV-2 is destroying global health and economy (1, 2). To date, SARS-CoV-2 has infected over 3 million people and caused more than 200,000 deaths. It forces much of the world to adopt a lockdown mode, causing staggering economic fallout and human suffering (https://www.cdc.gov/coronavirus/novel-coronavirus-2019.html). These numbers dwarf the impact of the related SARS coronavirus (SARS-CoV), which caused about 8,000 infections and 800 deaths (3, 4). Compared to SARS-CoV, many SARS-CoV-2 patients develop low levels of neutralizing antibodies and suffer prolonged illness (5–7). These clinical features indicate that SARS-CoV-2 evades the human immune surveillance more effectively than SARS-CoV does. When viruses evolve to escape immune surveillance, they often suffer reduced fitness and become less infectious (8–10). Yet SARS-CoV-2 remains highly infectious (11, 12). The combination of immune evasion and high infectivity may contribute to the wide spread of SARS-CoV-2. To curb SARS-CoV-2, it is important to uncover the molecular mechanisms that enable it to both evade immune surveillance and maintain high infectivity. Here, using biochemical and pseudovirus entry assays and SARS-CoV as a comparison, we investigate these mechanisms at an essential step of viral infection: the cell entry of SARS-CoV-2.

**Fig. 1.**
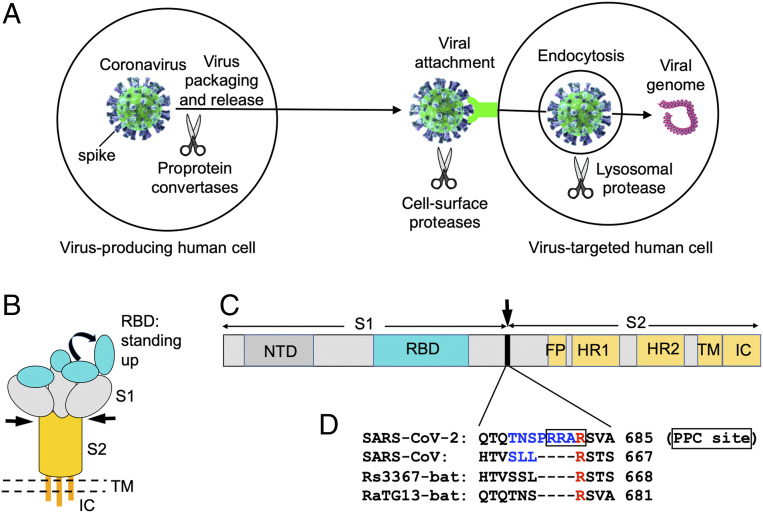
PPC motif in SARS-CoV-2 spike protein. (*A*) Different stages of coronavirus entry where host cellular proteases may activate coronavirus spikes. (*B*) Schematic drawing of the three-dimensional (3D) structure of coronavirus spike. S1, receptor-binding subunit; S2, membrane fusion subunit; TM, transmembrane anchor; IC, intracellular tail. (*C*) Schematic drawing of the 1D structure of coronavirus spike. NTD, N-terminal domain. FP (fusion peptide), HR1 (heptad repeat 1), and HR2 (heptad repeat 2) are structural units in coronavirus S2 that function in membrane fusion. (*D*) Sequence comparison of the spike proteins from SARS-CoV-2, SARS-CoV, and two bat SARS-like coronaviruses in a region at the S1/S2 boundary. Only SARS-CoV-2 spike contains a putative PPC motif—RRAR (residues in the box). The assumed PPC cleavage site is in front of the arginine residue labeled in red. The spike region mutated from SARS-CoV-2 sequence (TNSPRRA) to SARS-CoV sequence (SLL) is labeled in blue. GenBank accession numbers are QHD43416.1 for SARS-CoV-2 spike, AFR58740.1 for SARS-CoV spike, MG916901.1 for bat Rs3367 spike, and QHR63300.2 for bat RaTG13 spike.

Coronavirus entry into host cells is an important determinant of viral infectivity and pathogenesis (13, 14). It is also a major target for host immune surveillance and human intervention strategies (15, 16). To enter host cells, coronaviruses first bind to a cell surface receptor for viral attachment, subsequently enter endosomes, and eventually fuse viral and lysosomal membranes (13, 14) (Fig. 1*A*). A virus surface-anchored spike protein mediates coronavirus entry (Fig. 1 *B* and *C*). On mature viruses, the spike protein is present as a trimer, with three receptor-binding S1 heads sitting on top of a trimeric membrane fusion S2 stalk (Fig. 1*B*). The cell entry mechanism of SARS-CoV has been extensively studied. SARS-CoV S1 contains a receptor-binding domain (RBD) that specifically recognizes angiotensin-converting enzyme 2 (ACE2) as its receptor (17–19). The RBD constantly switches between a standing-up position for receptor binding and a lying-down position for immune evasion (20, 21) (Fig. 1*B*). Moreover, to fuse membranes, SARS-CoV spike needs to be proteolytically activated at the S1/S2 boundary, such that S1 dissociates and S2 undergoes a dramatic structural change (22, 23). These SARS-CoV entry-activating proteases include cell surface protease TMPRSS2 and lysosomal proteases cathepsins (22, 23) (Fig. 1*A*). These features of SARS-CoV entry contribute to its rapid spread and severe symptoms and high fatality rates of infected patients (24–26).

The past several months saw an explosion of studies on the cell entry mechanisms of SARS-CoV-2, sometimes with conflicting findings. Like SARS-CoV, SARS-CoV-2 also recognizes human ACE2 (hACE2) as its receptor (27–29). We recently determined the crystal structure of SARS-CoV-2 RBD complexed with hACE2, which revealed subtle but functionally important differences between SARS-CoV-2 and SARS-CoV in receptor recognition (30). These differences enable SARS-CoV-2 RBD to have a significantly higher hACE2 binding affinity than SARS-CoV RBD does (30). However, the cryo-electron microscopy (cryo-EM) structure of SARS-CoV-2 spike revealed that its RBD is mostly in the lying-down state (31, 32), a state associated with ineffective receptor binding. In addition, there have been conflicting reports on the hACE2-binding affinities of SARS-CoV-2 and SARS-CoV spikes (32–34).

In addition to receptor binding, protease activators for SARS-CoV-2 entry have been examined. It has been shown that TMPRSS2 and lysosomal proteases are both important for SARS-CoV-2 entry (33, 34). In avian influenza viruses, proprotein convertase (PPC) motif in the surface glycoprotein is a hallmark of high pathogenesis (35). However, although SARS-CoV-2 spike contains a PPC motif at the S1/S2 boundary, it was reported that PPC cleavage of the spike protein did not enhance SARS-CoV-2 entry into cells (31), challenging the well-established concept on the role of PPC motif. This raised questions about the role of PPC motif in SARS-CoV-2 entry.

Here we investigate the receptor binding and protease activations of SARS-CoV-2 spike, using SARS-CoV spike as a comparison. Our results identify important cell entry mechanisms of SARS-CoV-2 that potentially contribute to the immune evasion, cell infectivity, and wide spread of the virus. The findings reconcile conflicting recent reports on cell entry of SARS-CoV-2. By revealing the surprising strategies that SARS-CoV-2 adopts to infect humans while evading immune surveillance, the findings provide insight into possible intervention strategies targeting cell entry of the virus.

## Results

Through examining the sequence of SARS-CoV-2 spike, we identified a putative cleavage site for PPCs at the S1/S2 boundary (Fig. 1 *C* and *D*). Curiously, this putative PPC site is absent in the spikes of SARS-CoV and SARS-like bat coronaviruses. In this study, we investigated the role of PPC, along with other proteases, in SARS-CoV-2 entry. To this end, we established a pseudovirus entry assay for SARS-CoV-2. More specifically, replication-deficient lentiviruses were pseudotyped with SARS-CoV-2 spike (i.e., SARS-CoV-2 pseudoviruses) and used to enter target cells. This type of pseudovirus assay separates viral entry from other steps of the viral infection cycle (e.g., replication), enabling us to focus on the viral entry step that is mediated by SARS-CoV-2 spike. Three types of target cells were used: HeLa cells (human cervical cells) exogenously expressing hACE2, Calu-3 cells (human lung epithelial cells) endogenously expressing hACE2, and MRC-5 cells (human lung fibroblast cells) endogenously expressing hACE2.

**Fig. 2.**
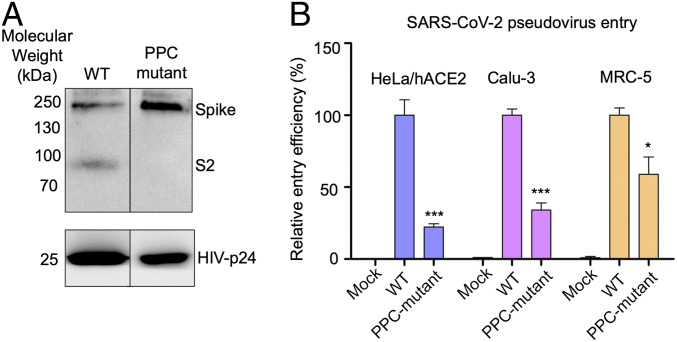
Role of PPC motif in SARS-CoV-2 spike-mediated cell entry. (*A*) Cleavage state of SARS-CoV-2 spike on the surface of pseudoviruses. Packaged SARS-CoV-2 pseudoviruses were subjected to Western blot analysis for detection of the cleavage state of SARS-CoV-2 spike. SARS-CoV-2 spike fragments were detected using anti-C9 antibody targeting the C-terminal C9 tag of the spike protein. (*Left*) Wild-type (WT) SARS-CoV-2 pseudoviruses. (*Right*) SARS-CoV-2 pseudoviruses where the PPC motif in the spike protein had been mutated to the corresponding sequence in SARS-CoV spike (see Fig. 1*D* for details). (*B*) SARS-CoV-2 pseudovirus entry into three types of target cells. The two types of pseudoviruses correspond to the pseudoviruses in *A*. Pseudovirus entry efficiency was characterized as luciferase signal accompanying entry. The entry efficiency of wild-type SARS-CoV-2 pseudoviruses was taken as 100%. Error bars indicate SD (*n* = 4). ***P < 0.001; *P < 0.05.

To detect the cleavage state of SARS-CoV-2 spike on the surface of pseudoviruses, we packaged SARS-CoV-2 pseudoviruses in HEK293T cells (human embryonic kidney cells) and performed Western blot on the pseudoviruses. The result showed that SARS-CoV-2 spike had been cleaved during viral packaging (Fig. 2*A*). We then mutated the putative PPC site in SARS-CoV-2 spike to the corresponding sequence in SARS-CoV spike; the mutant SARS-CoV-2 spike was no longer cleaved during viral packaging (Fig. 2*A*). Further, we performed pseudovirus entry assay using both wild-type SARS-CoV-2 pseudoviruses and PPC site mutant SARS-CoV-2 pseudoviruses. The result showed that SARS-CoV-2 pseudoviruses efficiently entered all three types of target cells (Fig. 2*B*). In contrast, the mutant SARS-CoV-2 pseudoviruses demonstrated significantly reduced efficiency in entering the same cells (Fig. 2*B*). The remaining cell entry of the mutant SARS-CoV-2 pseudoviruses was likely due to the activation from other host proteases that play partially overlapping and cumulative roles with PPCs (see below). Therefore, we have identified and confirmed the PPC cleavage site in SARS-CoV-2 spike, and shown that PPC cleavage of SARS-CoV-2 spike during viral packaging is critical for SARS-CoV-2 to enter three different types of target cells.

**Fig. 3.**
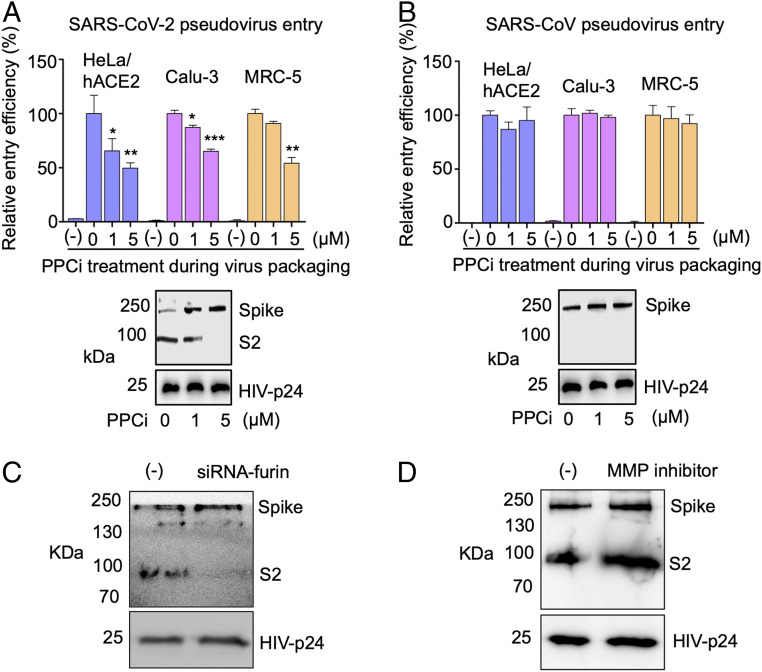
Effect of PPCs on SARS-CoV-2 spike-mediated cell entry. (*A*) SARS-CoV-2 pseudovirus entry into three types of target cells in the presence of PPCi. The pseudoviruses were packaged in the presence of different concentrations of PPCi before they were subjected to cell entry; (-) control: no pseudovirus was added. Also shown is the Western blot result of the corresponding pseudoviruses (packaged in the presence of different concentrations of PPCi). The entry efficiency of SARS-CoV-2 pseudoviruses without any treatment was taken as 100%. Error bars indicate SD (*n* = 4). ***P < 0.001; **P 0.01; *P < 0.05. (*B*) SARS-CoV pseudovirus entry into three types of target cells in the presence of PPCi. The experiments were performed in the same way as in *A*, except that SARS-CoV spike replaced SARS-CoV-2 spike in pseudoviruses. The entry efficiency of SARS-CoV pseudoviruses without any treatment was taken as 100%. (*C*) Western blot result of SARS-CoV-2 pseudoviruses packaged in cells treated with siRNA. (*Left*) Pseudoviruses packaged in cells treated with siRNA-negative control. (*Right*) Pseudoviruses packaged in cells treated with furin-targeting siRNA. (*D*) Western blot result of SARS-CoV-2 pseudoviruses packaged in cells treated with MMP inhibitor. (*Left*) Pseudoviruses packaged in cells not treated with MMP inhibitor. (*Right*) Pseudoviruses packaged in cells treated with MMP inhibitor.

To provide further evidence for the role of prior PPC cleavage in SARS-CoV-2 entry, we treated HEK293T cells with PPC inhibitor (PPCi) during packaging of wild-type SARS-CoV-2 pseudoviruses, and then subjected the PPCi-treated SARS-CoV-2 pseudoviruses to entry into the aforementioned three types of target cells. The result showed that PPCi treatment inhibited PPC cleavage of SARS-CoV-2 spike on pseudoviruses, and that the PPCi-treated SARS-CoV-2 pseudoviruses demonstrated significantly reduced cell entry efficiency (Fig. 3*A*). In comparison, SARS-CoV spike was not cleaved during packaging of SARS-CoV pseudoviruses, and PPCi treatment during virus packaging had no effect on the subsequent cell entry process (Fig. 3*B*). These results further confirm that the efficiency of SARS-CoV-2 entry into target cells can be enhanced by the prior PPC cleavage of the SARS-CoV-2 spike during viral packaging, a contrast to SARS-CoV whose cell entry does not depend on PPC preactivation.

Since the PPCi used above is a broad-spectrum PPCi, we further investigated which specific PPC activates SARS-CoV-2 spike using small interfering RNA (siRNA) assay. To this end, we packaged SARS-CoV-2 pseudoviruses in HEK293T cells that were treated with furin-targeting siRNA. Furin was selected in our study because it is the prototypic PPC and it preactivates the entry of many other viruses, including some coronaviruses (22, 23). The result showed that, after furin-targeting siRNA treatment, the spike molecules on the packaged SARS-CoV-2 pseudoviruses were intact (Fig. 3*C*), revealing that furin is the PPC that preactivates SARS-CoV-2 spike. To rule out the possibility that furin-dependent activation of matrix metalloproteinases (MMPs) led to indirect activation of SARS-CoV-2 spike, we treated HEK293T cells with MMP inhibitor during packaging of SARS-CoV-2 pseudoviruses. The result showed that, after MMP inhibitor treatment, the spike molecules on the packaged SARS-CoV-2 pseudoviruses were still cleaved (Fig. 3*D*), demonstrating that MMP is not involved in the activation of SARS-CoV-2 spike. Taken together, these findings show that furin is the PPC that preactivates SARS-CoV-2 spike (1, 2).

**Fig. 4.**
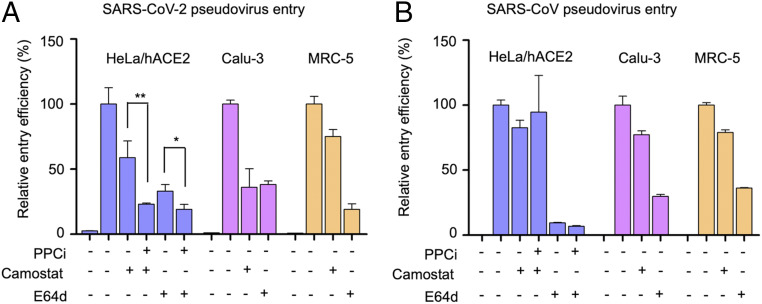
Effect of other protease inhibitors on SARS-CoV-2 entry. (*A*) SARS-CoV-2 pseudovirus entry into three types of target cells in the presence of protease inhibitors. For pseudoviruses treated with PPCi, the pseudoviruses were packaged in the presence of PPCi (5 µM) before they were subjected to cell entry. For pseudoviruses treated with TMPRSS2 inhibitor camostat or lysosomal protease inhibitor E64d, pseudovirus entry was performed in the presence of camostat (50 µM) or E64d (50 µM). The cleavage state of SARS-CoV-2 spike was the same as in Fig. 3*A* (5 µM PPCi condition). The entry efficiency of SARS-CoV-2 pseudoviruses without any treatment was taken as 100%. Error bars indicate SD (*n* = 4). ***P < 0.001; *P < 0.05. (*B*) SARS-CoV pseudovirus entry into three types of target cells. The treatments were done in the same way as in *A*.

To investigate the role of other proteases in SARS-CoV-2 entry, we performed pseudovirus entry assay in the presence of inhibitors that specifically target these other proteases. First, SARS-CoV-2 pseudovirus entry into all three types of target cells was reduced in the presence of TMPRSS2 inhibitor camostat (Fig. 4*A*), suggesting that these cells endogenously express TMPRSS2 and that these TMPRSS2 molecules activate SARS-CoV-2 entry. Second, SARS-CoV-2 pseudovirus entry into all three types of target cells was reduced in the presence of lysosomal cathepsin inhibitor E64d (Fig. 4*A*). Hence, lysosomal cathepsins activate SARS-CoV-2 entry. Similarly, SARS-CoV entry can also be activated by TMPRSS2 and lysosomal cathepsin (Fig. 4*B*). Moreover, prior treatment of pseudovirus-packaging cells with PPCi, combined with treatment of pseudovirus-targeted cells with either camostat or E64d, further reduced the efficiency of SARS-CoV-2 pseudovirus entry into HeLa cells (Fig. 4*A*). Thus, TMPRSS2 and lysosomal cathepsins both have cumulative effects with furin on activating SARS-CoV-2 entry. In contrast, neither camostat nor E64d has cumulative effects with PPCi on activating SARS-CoV entry (Fig. 4*B*). Overall, these results demonstrate that cell surface proteases and lysosomal proteases can both activate SARS-CoV-2 entry; in addition, furin and these other proteases have cumulative effects on activating SARS-CoV-2 entry.

**Fig. 5.**
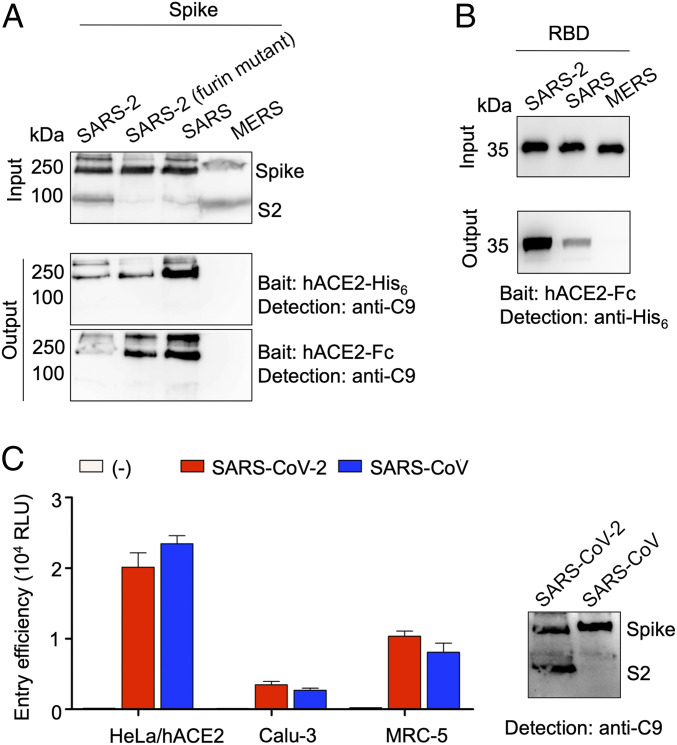
Comparison of receptor binding affinity and cell entry efficiency of SARS-CoV-2 and SARS-CoV. (*A*) Spike pull-down assay using hACE2 as the bait and cell-associated coronavirus spike molecules as the targets. (*Top*) Cell-expressed coronavirus spike molecules including SARS-CoV-2 spike, SARS-CoV-2 spike containing a mutant furin site as in Fig. 2*A*, SARS-CoV spike, and MERS-CoV spike. These spike molecules all contain a C-terminal C9 tag. (*Middle*) Pull-down result using His_6_-tagged hACE2. (*Bottom*) Pull-down result using Fc-tagged hACE2. (*B*) RBD pull-down assay using Fc-tagged hACE2 as the bait and soluble coronavirus RBDs as the targets. These RBD molecules all contain a C-terminal His_6_ tag. (*C*) (*Left*) Entry of SARS-CoV-2 and SARS-CoV pseudoviruses into three types of target cells. (*Right*) Western blot of SARS-CoV-2 and SARS-CoV pseudoviruses used in the cell entry assay.

Having examined the role of furin in cleaving SARS-CoV-2 spike and preactivating SARS-CoV-2 entry, we next compared the hACE2-binding affinities of SARS-CoV-2 and SARS-CoV spikes. To this end, we performed a protein pull-down assay, using recombinant hACE2 as the bait and cell surface-expressed SARS-CoV-2 and SARS-CoV spikes as the targets. To eliminate any potential effect of furin cleavage on SARS-CoV-2 spike’s binding of hACE2, we also included SARS-CoV-2 spike with its furin site mutated. For cross-validation, we used hACE2 with two different tags, His_6_ tag and Fc tag. The result showed that, compared to SARS-CoV spike, SARS-CoV-2 spike binds to hACE2 with lower affinity (Fig. 5*A*). This result is different from our recent report that SARS-CoV-2 RBD binds to hACE2 with significantly higher affinity than SARS-CoV RBD does, which was detected using surface plasmon resonance (SPR) (30). To ensure that the above discrepancy was not due to different detection methods, we performed protein pull-down assay using recombinant hACE2 as the bait and soluble SARS-CoV-2 and SARS-CoV RBDs as the targets. The result showed that SARS-CoV-2 RBD binds to hACE2 with significantly higher affinity than SARS-CoV RBD does (Fig. 5*B*), confirming our recent SPR result. Therefore, whereas SARS-CoV-2 RBD has higher hACE2 binding affinity than SARS-CoV RBD, SARS-CoV-2 spike has lower hACE2 binding affinity than SARS-CoV spike.

Finally, we directly compared the cell entry efficiency of SARS-CoV-2 and SARS-CoV pseudoviruses. Similar to recent studies (31, 34), we calibrated pseudovirus entry efficiency against expression levels of spikes. Moreover, taking into account that part of SARS-CoV-2 spike molecules had been cleaved during pseudovirus packaging, we used the total amount of uncleaved and cleaved spike molecules to calibrate SARS-CoV-2 pseudovirus entry, while using the uncleaved spike molecules to calibrate SARS-CoV pseudovirus entry. The result showed that SARS-CoV-2 and SARS-CoV pseudoviruses entered all three types of target cells with similar efficiency (Fig. 5*C*), which is consistent with two recent studies (31, 34).

## Discussion

With mounting infections, fatalities, and economic losses caused by SARS-CoV-2, it is imperative that we understand the cell entry mechanisms of SARS-CoV-2. However, recent studies have presented puzzling and sometimes conflicting findings on how SARS-CoV-2 enters cells, raising pressing scientific questions (30–32, 34). For example, which virus binds to hACE2 more tightly, SARS-CoV-2 or SARS-CoV? What is the role of furin in SARS-CoV-2 entry? How does SARS-CoV-2 successfully evade human immune surveillance while maintaining its high cell infectivity? The current study addresses these questions by detailing the cell entry mechanisms of SARS-CoV-2.

**Fig. 6.**
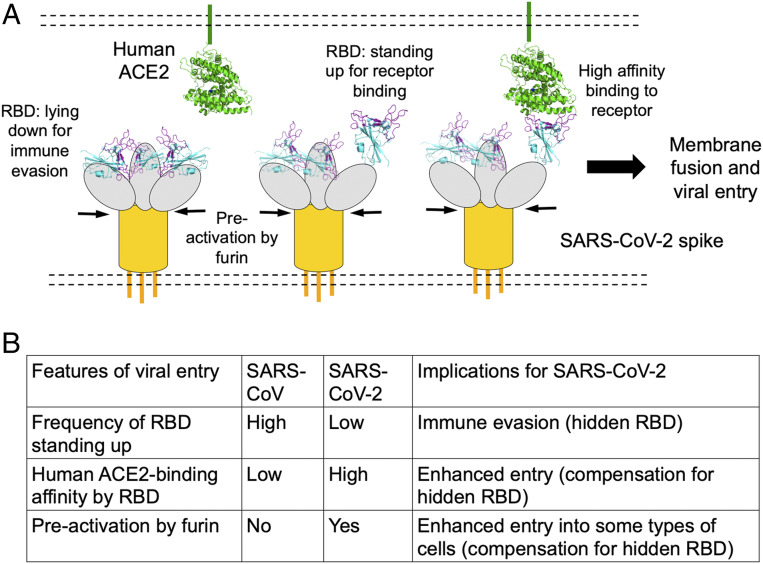
Summary of cell entry mechanisms of SARS-CoV-2. (*A*) A schematic view of three unique features of SARS-CoV-2 entry: hidden RBD in the spike for immune evasion, RBD’s high hACE2 binding affinity for efficient entry, and furin preactivation of the spike for enhanced entry into some cells. (*B*) Implications of the cell entry mechanisms of SARS-CoV-2.

Receptor recognition is an important determinant of coronavirus infection and pathogenesis. It is also one of the most important targets for host immune surveillance and human intervention strategies. The current study and other recent studies have revealed two patterns of results on the hACE2 binding affinity of SARS-CoV-2. First, with regard to the RBD, SARS-CoV-2 RBD has significantly higher hACE2 binding affinity than SARS-CoV RBD does. This was shown in our recent study using SPR assay as well as structural and mutagenesis analyses (30). In addition, using protein pull-down assay, the current study confirmed that SARS-CoV-2 RBD has higher hACE2 binding affinity than SARS-CoV RBD does. Second, despite the potency of its RBD’s binding to hACE2, the entire SARS-CoV-2 spike does not bind to hACE2 any more strongly than SARS-CoV spike does. Using protein pull-down assay, the current study showed that SARS-CoV-2 spike binds to hACE2 less strongly than SARS-CoV spike does. Another study using flow cytometry assay yielded similar results (34). A third study using Blitz assay showed that SARS-CoV-2 and SARS-CoV spikes have similar hACE2 binding affinities (31). Note that the hACE2 binding affinities of SARS-CoV RBD and SARS-CoV-2 spike should not be compared directly with each other (32). These findings therefore present a paradoxical pattern of results: Although SARS-CoV-2 RBD has higher hACE2 binding affinity than SARS-CoV RBD, its spike has hACE2 binding affinity comparable to or lower than SARS-CoV spike. These contrasting patterns between the RBD and the entire spike are particularly compelling in the current study because they were observed using the same method and under the same testing conditions. The dynamic state of the RBD in coronavirus spikes may explain this paradox. The RBD in coronaviruses can be in either a standing-up state, which enables receptor binding, or a lying-down state, which does not bind to the host receptors (20, 21). Cryo-EM studies have shown that, in SARS-CoV spike, the RBD is mostly in the standing-up state (20, 21); however, in SARS-CoV-2 spike, the RBD is mostly in the lying-down state (31, 32). Therefore, compared to SARS-CoV, although SARS-CoV-2 RBD has higher hACE2 binding affinity, it is less accessible, resulting in comparable or lower hACE2 binding affinity for SARS-CoV-2 spike (Fig. 6*A*).

To maintain its high infectivity while keeping its RBD less accessible, SARS-CoV-2 relies on a second strategy—host protease activation. Host protease activation is a significant determinant of coronavirus infection and pathogenesis, and a significant target for host immune surveillance and human intervention strategies. Using a combination of mutagenesis, protease inhibitors, and siRNA approaches, here we showed that furin preactivation enhances SARS-CoV-2 pseudovirus entry into different types of hACE2-expressing cell lines, including lung epithelial and lung fibroblast cell lines. We also showed that cell surface protease TMPRSS2 and lysosomal cathepsins activate SARS-CoV-2 pseudovirus entry and that both TMPRSS2 and cathepsins have cumulative effects with furin on SARS-CoV-2 entry. In comparison, SARS-CoV pseudovirus entry is activated by TMPRSS2 and cathepsins, but not furin. Furin preactivation allows SARS-CoV-2 to be less dependent on target cells, enhancing its entry into some target cells, particularly cells with relatively low expressions of TMPRSS2 and/or lysosomal cathepsins. This has also been observed with furin-preactivated avian influenza viruses (32). However, a recent study showed that furin preactivation enhances SARS-CoV-2 pseudovirus entry into BHK cells (baby hamster kidney fibroblast cells), but reduces SARS-CoV-2 pseudovirus entry into Vero cells (African green monkey kidney epithelial cells) (31). These seemingly conflicting results can be explained by how coronavirus entry is regulated by proteases. Protease activation of coronavirus spikes potentially leads to the final structural change of coronavirus S2 needed for membrane fusion; this process is irreversible and needs to be tightly regulated (13). Indeed, it has been shown that, on SARS-CoV-2 virus particles, many spike molecules have already undergone the final structural change (36). Hence, in principle, virus particles preactivated by furin may have unchanged or reduced entry efficiency in some types of cells with high expressions of TMPRSS2 and/or lysosomal proteases; this may particularly be the case in vitro for virus particles that are not fresh, as the final conformational change of spike molecules may occur slowly spontaneously or be facilitated by environmental factors (e.g., high temperature, physical force, or some chemicals) (37). Overall, furin preactivation can facilitate SARS-CoV-2 to enter some types of cells (particularly those with low expressions of TMPRSS2 and/or lysosomal cathepsins) (Fig. 6*A*).

The cell entry mechanisms of SARS-CoV-2 have implications for understanding clinical features of coronavirus disease 2019 (COVID-19) (Fig. 6*B*). The hidden RBD can evade immune surveillance, potentially leading to insufficient immune responses and prolonged recovery time. Granted, there are other immune evasion strategies for coronaviruses. For example, some coronavirus nonstructural proteins can help evade the host innate immune responses (38, 39). Importantly, viruses commonly hide their RBD or other critical parts of their spike proteins from host adaptive immune responses using two main strategies (40). The first is conformational masking, where viruses conceal their RBDs in locations like canyons (as in the case of picornaviruses) (41) or recessed pockets (as in the case of HIV) (42). The second is glycan shielding, where viruses conceal critical parts of their spike proteins behind glycan clusters (as in the case of HIV, Ebola virus, and hepatitis C virus) (43). Our finding about the discrepancy in hACE2 binding affinity between SARS-CoV-2 RBD and spike, combined with other groups’ observation of the lying-down RBD in SARS-CoV-2 spike, suggests that the hidden RBD contributes to the immune evasion of SARS-CoV-2 as one of the conformational masking strategies. Indeed, a recent study showed that SARS-CoV RBD-induced mouse sera bind SARS-CoV-2 RBD with high affinity, but poorly neutralize SARS-CoV-2 pseudovirus entry into host cells; in contrast, the same sera bind SARS-CoV RBD with high affinity and neutralize SARS-CoV pseudovirus entry potently (44). This result shows that immune surveillance recognizes hidden RBD less well than exposed RBD. However, hidden RBD may lead to poor recognition of the host receptor and inefficient entry into host cells. SARS-CoV-2 overcomes this problem by evolving an RBD with high hACE2 binding affinity and a furin motif that allows its spike to be preactivated. The end result is that the overall entry efficiencies of SARS-CoV-2 and SARS-CoV pseudoviruses are comparable.

Understanding the cell entry mechanism of SARS-CoV-2 can inform intervention strategies. The RBD is the most immunogenic region of the whole spike (15, 45). Hence, the hidden RBD of SARS-CoV-2 presents a major challenge to both vaccination and antibody drug therapy due to the limited access of neutralizing antibodies to the target. Correspondingly, there are several approaches for intervention strategies, with some caveats. First, antibody drugs can be developed to bind to the RBD very tightly, preferably with both a high *k*_on_ rate and a low *k*_off_ rate, such that, during the limited exposure of RBD, the drugs can latch onto the RBD quickly and keep a strong hold on it. It was recently shown that recombinant ACE2 can inhibit SARS-CoV-2 infection in artificial human tissues (46), suggesting that blocking the RBD is feasible. Thus, an antibody drug with significantly higher RBD binding affinity than ACE2 can dominate over cell surface ACE2 in latching onto the RBD, blocking viral attachment. Second, RBD vaccines can be developed. Because neutralizing antibodies elicited by RBD vaccines may have limited access to the RBD, structure-guided engineering will be needed to significantly enhance the efficacy of RBD vaccines (45). Third, vaccines and drugs can be developed to target the membrane fusion S2 subunit. The success of this approach for vaccine development, however, may be limited because the S2 subunit is less immunogenic than the RBD (15). Last, the cell entry process of SARS-CoV-2 can be blocked using inhibitors that target the protease activators (47). Because SARS-CoV-2 uses several cellular proteases as entry activators, inhibitor mixtures against multiple protease activators would be needed to achieve satisfactory outcome. This approach will need to consider side effects when these drugs target host proteins. The sophisticated cell entry mechanisms of SARS-CoV-2 pose significant challenges, but also illuminate multiple intervention strategies that target cell entry of the virus.

## Materials and Methods

### Cell Line and Plasmids.

HEK293T, HeLa, Calu-3, and MRC-5 cells were obtained from the American Type Culture Collection and cultured in Dulbecco’s modified Eagle medium supplemented with 10% fetal bovine serum, 2 mM L-glutamine, 100 units/mL penicillin, and 100 µg/mL streptomycin (Life Technologies).

Full-length SARS-CoV-2 spike (GenBank accession number QHD43416.1), SARS-CoV Spike (GenBank accession number AFR58740.1), MERS-CoV spike (GenBank accession number AFS88936.1), and human ACE2 (GenBank accession number NM_021804) were synthesized (GenScript Biotech) and subcloned into the pcDNA3.1(+) vector (Life Technologies) with a C-terminal C9 tag. SARS-CoV-2 RBD (residues 319 to 535), SARS-CoV RBD (residues 306 to 521), MERS-CoV RBD (residues 367 to 588), and human ACE2 peptidase domain (residues 1 to 615) were subcloned into pFastBac vector (Life Technologies) with an N-terminal honey bee melittin signal peptide and a C-terminal His_6_ tag. For human ACE2 peptidase domain, a construct was also made containing a C-terminal Fc tag instead of the C-terminal His_6_ tag.

### Protein Expression and Purification.

All of the proteins were expressed in sf9 insect cells using the Bac-to-Bac system (Life Technologies). Briefly, His_6_-tagged proteins were harvested from cell culture medium, and were purified sequentially on Ni-NTA column and Superdex200 gel filtration column (GE Healthcare) as described previously (30). The Fc-tagged protein was purified in the same way, except that protein A column replaced Ni-NTA column (30). Purified proteins were stored in a buffer containing 20 mM Tris pH7.2 and 200 mM NaCl for later use.

### Coronavirus Spike-Mediated Pseudovirus Entry Assay.

Retroviruses pseudotyped with SARS-CoV-2 spike or SARS-CoV spike were generated in HEK293T cells, and pseudovirus entry assay was performed as previously described (48). Briefly, HEK293T cells were cotransfected with a plasmid carrying an Env-defective, luciferase-expressing HIV-1 genome (pNL4-3.luc.R-E-) and pcDNA3.1(+) plasmid encoding one of the indicated spikes. Pseudoviruses were harvested 72 h after transfection, and were used to enter target cells. Six hours after incubation with pseudoviruses, cells were transferred to fresh medium. After another 66 h, cells were washed and lysed for detection of luciferase signal (relative luciferase units or RLU). Target cells for pseudovirus entry assay included HeLa cells exogenously expressing human ACE2, and Calu-3 and MRC-5 cells endogenously expressing human ACE2.

For pseudoviruses treated with PPCi or matrix MMP inhibitor, PPCi chloromethylketone (Enzo Life Sciences) or MMP inhibitor batimastat (Sigma-Aldrich) was added to the medium at indicated concentrations 6 h after transfection for pseudovirus packaging began. Pseudoviruses were harvested after an additional incubation time of 66 h. Pseudoviruses were then used to enter target cells.

For pseudoviruses treated with siRNA, siRNA furin and siRNA negative control (Thermo Fisher Scientific) were transfected separately into HEK293T cells 6 h after transfection for pseudovirus packaging began. Pseudoviruses were harvested after an additional incubation time of 66 h. Pseudoviruses were then subjected to Western blot analysis.

For pseudoviruses treated with other protease inhibitors, target cells were pretreated with camostat (50 µM) (Sigma-Aldrich) or E64d (50 µM) (Sigma-Aldrich) for 1 h and then subjected to pseudovirus entry assay as described above.

### Protein Pull-Down Assay.

Protein pull-down assay was performed using a Dynabeads immunoprecipitation kit (Invitrogen) as previously described (30). Briefly, 80 µL of Dynabeads, either for His_6_-tagged proteins or for Fc-tagged proteins, were washed with phosphate-buffered saline (PBS) buffer and then were incubated with either 5 µg hACE2-His_6_ (human ACE2 with a C-terminal His_6_ tag) or 5 µg hACE2-Fc (human ACE2 with a C-terminal Fc tag), respectively, on a roller at room temperature for 30 min. Subsequently, hACE2-bound beads were washed three times with 1 mL of PBS buffer plus 0.05% Tween-20 (PBST) on a roller for 10 min and then were aliquoted into different tubes for later use. To prepare cell-associated coronavirus spike protein, HEK293T cells were transfected with pcDNA3.1(+) plasmid encoding coronavirus spike (containing a C-terminal C9 tag); 48 h after transfection, the spike-expressing cells were lysed using a sonicator in assay buffer and centrifuged at 12,000 × *g* for 2 min. The supernatants containing solubilized SARS-CoV-2 spike (for spike pull-down assay) or purified recombinant coronavirus RBDs (for RBD pull-down assay) were incubated with the hACE2-bound beads in 2-mL tubes (spike or RBD was in excess of hACE2) on a roller at room temperature for 1 h. Then beads were washed three times with PBST buffer, and the bound proteins were eluted using elution buffer. The samples were then subjected to Western blot analysis and detected using an anti-C9 tag antibody or anti-His tag antibody.

### Statistic Analysis.

All experiments were repeated at least four times. Statistical analyses were performed using *t* tests. A *P* value < 0.05 was considered statistically significant; ***P < 0.001. **P < 0.01. *P < 0.05.

### Data Availability Statement.

All data discussed in the paper are available in Dataset S1.

## Supplementary Material

Supplementary File
